# Creation of Golden Gate constructs for gene doctoring

**DOI:** 10.1186/s12896-020-00648-5

**Published:** 2020-10-07

**Authors:** Nicholas M. Thomson, Chuanzhen Zhang, Eleftheria Trampari, Mark J. Pallen

**Affiliations:** 1 Quadram Institute Bioscience, Norwich Research Park, Norwich, Norfolk NR4 7UQ UK; 2 National Risk Assessment Laboratory for Antimicrobial Resistance of Animal Original Bacteria, College of Veterinary Medicine, South China Agricultural University, Guangzhou, 510642 China; 3 Guangdong Key Laboratory for Veterinary Drug Development and Safety evaluation, College of Veterinary Medicine, South China Agricultural University, Guangzhou, 510642 China; 4 School of Biological Sciences, University of East Anglia, Norwich Research Park, Norwich, Norfolk NR4 7TU UK; 5 School of Veterinary Medicine, University of Surrey, Daphne Jackson Road, Guildford, Surrey GU2 7AL UK

**Keywords:** Gene doctoring, Recombineering, Golden Gate assembly, Mutagenesis, Enterobacteria, Chromosome, Insertion, Deletion

## Abstract

**Background:**

Gene doctoring is an efficient recombination-based genetic engineering approach to mutagenesis of the bacterial chromosome that combines the λ-Red recombination system with a suicide donor plasmid that is cleaved in vivo to generate linear DNA fragments suitable for recombination. The use of a suicide donor plasmid makes Gene Doctoring more efficient than other recombineering technologies. However, generation of donor plasmids typically requires multiple cloning and screening steps.

**Results:**

We constructed a simplified acceptor plasmid, called pDOC-GG, for the assembly of multiple DNA fragments precisely and simultaneously to form a donor plasmid using Golden Gate assembly. Successful constructs can easily be identified through blue-white screening. We demonstrated proof of principle by inserting a gene for green fluorescent protein into the chromosome of *Escherichia coli*. We also provided related genetic parts to assist in the construction of mutagenesis cassettes with a tetracycline-selectable marker.

**Conclusions:**

Our plasmid greatly simplifies the construction of Gene Doctoring donor plasmids and allows for the assembly of complex, multi-part insertion or deletion cassettes with a free choice of target sites and selection markers. The tools we developed are applicable to gene editing for a wide variety of purposes in *Enterobacteriaceae* and potentially in other diverse bacterial families*.*

## Background

Over 10 years ago, Lee et al. [1] developed the gene doctoring approach for efficient mutagenesis of *Escherichia coli*, targeting the λ-Red recombination system [2] to a mutation cassette flanked by homologous regions and released from a suicide donor plasmid by the I-SceI meganuclease. Delivery of the mutagenesis cassette from a plasmid rather than as a linear DNA fragment protects the DNA from attack by host nucleases and leads to higher-efficiency recombination. Since then, the gene doctoring technique has been refined [3] and used in other bacterial species, including *Salmonella enterica* [4], *Pseudomonas putida* [5], and *Klebsiella pneumoniae* [6]. However, construction of donor plasmids suitable for gene doctoring typically relies on multiple, sequential steps.

Golden Gate assembly [7, 8] provides a method for one-step combinatorial DNA assembly which, we hoped, could simplify construction of mutagenesis cassettes for gene doctoring. We therefore set about creating a plasmid into which multiple genetic elements could be inserted via a single Golden Gate reaction to produce a Gene Doctoring donor plasmid. Our plasmid, pDOC-GG, was based on pDOC-K from the original Gene Doctoring toolkit, but incorporated five changes to streamline the workflow from cloning to mutagenesis:

1. To improve compatibility with existing Golden Gate libraries*,* we changed the antibiotic resistance marker on the donor plasmid from *bla* (ampicillin resistance) to *aph (3′)-Ia* (kanamycin resistance).
2. We replaced the kanamycin resistance cassette of pDOC-K with a *lac*Zα drop-out cassette flanked by BsaI restriction sites to create a Golden Gate cloning region. Following cloning and transformation, successful colonies can be identified by blue-white screening [9] based upon loss of the drop-out cassette.
3. We removed a BsaI site from the pDOC-K backbone, between the HR2 cloning region and the *rrnB* T1 terminator. This prevents interference from off-target cleavage of the plasmid by BsaI during cloning.
4. We removed both multiple cloning sites so that only a minimal 34 bp FRT site will remain in the chromosome following removal of a resistance marker [10].
5. We reduced the size of the plasmid backbone, to help avoid problems with transformation and stability of a large plasmid prior to mutagenesis.

## Results

**Fig. 1:**
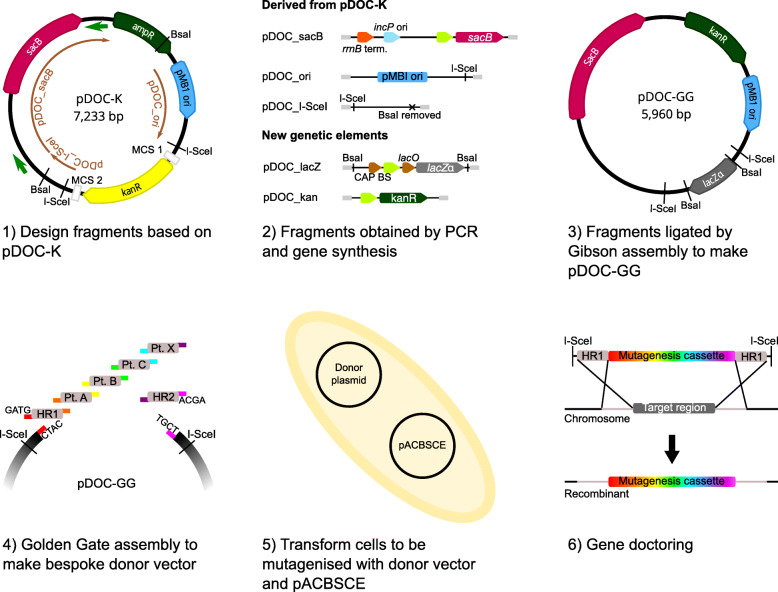
Construction of pDOC-GG and its use to produce Gene Doctoring donor plasmids and perform chromosomal modifications. The first three stages describe the production of pDOC-GG. 1) Three DNA fragments (brown arrows) were designed, based on pDOC-K, to form the backbone of pDOC-GG. Significant features of pDOC-K are displayed. White boxes represent multiple cloning sites (MCS) 1 and 2; dark green arrows represent PCR primers sacB_GA_F and sacB_GA_R used to obtain fragment pDOC_sacB. Other fragments were produced by chemical synthesis. 2) Full features of the three fragments designed from pDOC-K and two further fragments to introduce new genetic elements. Light green arrows represent promoters. 3) Significant features of pDOC-GG, which was produced by Gibson assembly of the five fragments. The last three stages describe the construction of mutagenesis cassettes in pDOC-GG, and use of the resulting donor plasmid for Gene Doctoring. 4) BsaI overhangs (coloured boxes) for homologous regions (HR) 1 and 2 should be complementary to the CTAC and ACGA overhangs generated on pDOC-GG. Multiple intervening parts (Pt. A – X) are assembled sequentially by design of appropriate complementary BsaI overhangs. 5) and 6) The donor plasmid is used for Gene Doctoring as described by Lee et al. [1]

By a combination of PCR and chemical synthesis, we obtained five linear DNA fragments encoding the necessary elements for pDOC-GG. Three fragments were derived from pDOC-K, while the other two encoded the new *aph (3′)-Ia* kanamycin resistance gene and the *lacZα* drop-out cassette. We then constructed pDOC-GG by ligating these fragments using Gibson assembly (Fig. 1) [11].

To construct a Gene Doctoring donor plasmid, a mutagenesis cassette is built from blocks of DNA with defined functions (known as genetic parts in the synthetic biology terminology) by Golden Gate assembly, replacing the *lacZα* cassette. Each mutagenesis cassette should begin with the first homologous region (HR1), ligated at its 5′ end to a CTAC BsaI overhang on pDOC-GG, and end with the second homologous region (HR2), ligated at its 3′ end to an ACGA overhang on pDOC-GG. Large numbers of intervening genetic parts can be assembled by designing them with sequential, overlapping BsaI overhangs. After verifying the correct sequence of the mutagenesis cassette, the donor plasmid can then be used for Gene Doctoring as described by Lee et al. [1].

In verifying the full sequence of pDOC-GG, we identified two inconsistencies from the previously published pDOC-K sequence: a deletion of a T at bp 4268 and a 23 bp insertion starting at bp 4336 (both numbered and with reference to pDOC-K). Since neither of the variations are in a coding or regulatory region, and since they are present in the original, widely used, Gene Doctoring plasmid we have no reason to suspect that they are significant for the function of the plasmid. We confirmed the function of pDOC-GG in *E. coli* by growth in the presence of kanamycin and generation of blue colonies in the presence of 5-bromo-4-chloro-3-indolyl-β-D-galactopyranoside (X-gal) and isopropyl β-D-1-thiogalactopyranoside (IPTG).

**Fig. 2:**
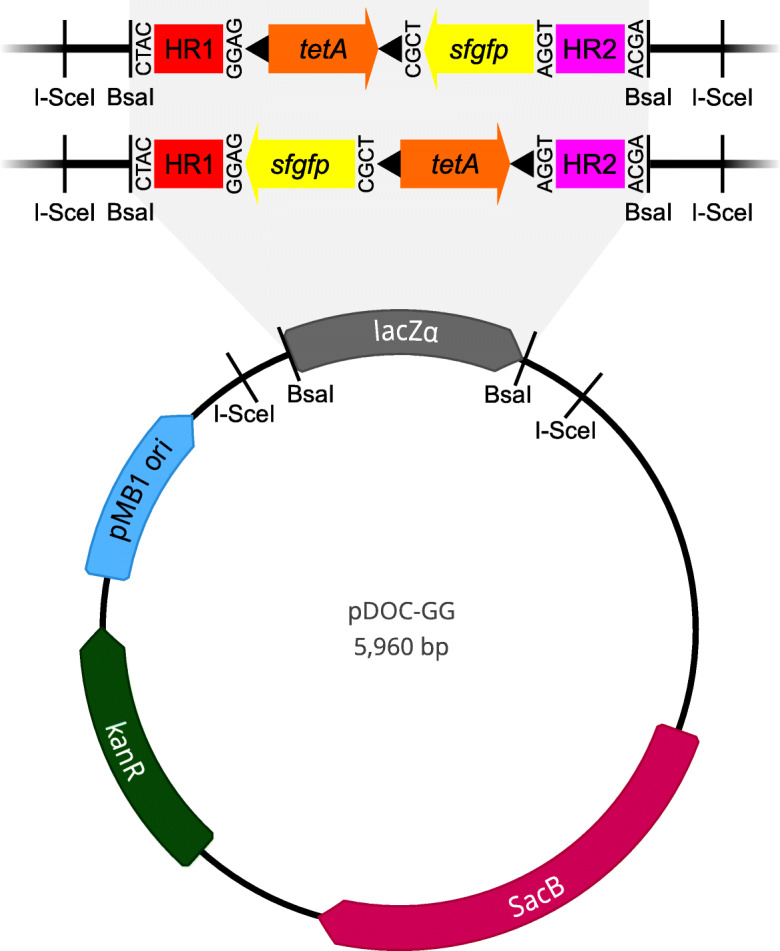
Schematic overview of the design of Gene Doctoring mutagenesis cassettes in pDOC-GG, using our donor vector for insertion of *sfgfp* into the *glmS* site of *E. coli* as an example. Golden Gate assembly is used to replace the *lacZα* cassette with the DNA sequence that will form the linear recombination fragment upon digestion with I-SceI in vivo. The top mutagenesis cassette shows the sequence that we assembled, with tetA upstream of *sfgfp*. The bottom cassette shows an alternative design, using our second *tetA* genetic part to locate the FRT scar sequence downstream of *sfgfp*. Mutagenesis cassettes are always flanked by homologous regions (HR1 and HR2) to direct them to the correct location in the chromosome. By designing appropriate 4-bp BsaI overhangs (vertical text), the number and order of the DNA parts can be varied as desired

We also produced two plasmid-borne genetic parts consisting of a tetracycline resistance cassette (*tetA* gene with a promoter and terminator) with different BsaI overhangs, flanked by FRT sites, in an Amp^R^ variant of mUAV [12]. These simplify the design of chromosomal insertions with the scar sequence on either side of the insertion to minimise polar effects on surrounding genes after removal of the *tetA* cassette by Flp recombinase (Fig. 2) [13].

To test the function of pDOC-GG for cloning and mutagenesis, we used it to insert *sfgfp*, encoding superfolder green fluorescent protein (sfGFP) into a highly conserved chromosomal locus downstream of the *glmS* gene of *E. coli* (referred to here as the *glmS* site), where fitness impacts from disruption are known to be minimal in multiple species [14–16]. Our mutagenesis cassette used one of the new *tetA* genetic parts immediately after HR1, followed by *sfgfp* in the reverse orientation relative to *glmS* (Fig. 2). Following Golden Gate cloning, we obtained almost exclusively white colonies on plates containing kanamycin, tetracycline, X-gal and IPTG. The white colonies fluoresced green under a blue-light lamp and developed a green colour following prolonged incubation (~ 24 h), confirming the function of *sfgfp*. All the green colonies (6/6) picked for screening by colony PCR with primers pDOC-K_F and pDOC-K_R had inserts of the expected length.

We purified the donor plasmid from one of the successful colonies and used it, together with pACBSCE [1], for mutagenesis by Gene Doctoring. We confirmed successful integration of *tetA* and *sfgfp* into the chromosome by colony PCR using primers glmS_screen_F and glmS_screen_R (Table 2). Out of 50 randomly chosen colonies screened, 43 (86%) grew on LB agar with tetracycline and sucrose and gave a colony PCR product of the expected size in agarose gel electrophoresis (Supplementary Figure 1). This compares favourably with the 90–93% integration efficiency (depending on the gene) reported for *E. coli* MG1655 by Lee et al [1]. The slight reduction in efficiency in our experiments is probably due to the larger mutagenesis fragment required for insertion of *sfgfp* plus a selection cassette rather than only the selection cassette required for the gene knockouts performed in the original paper.

**Fig. 3:**
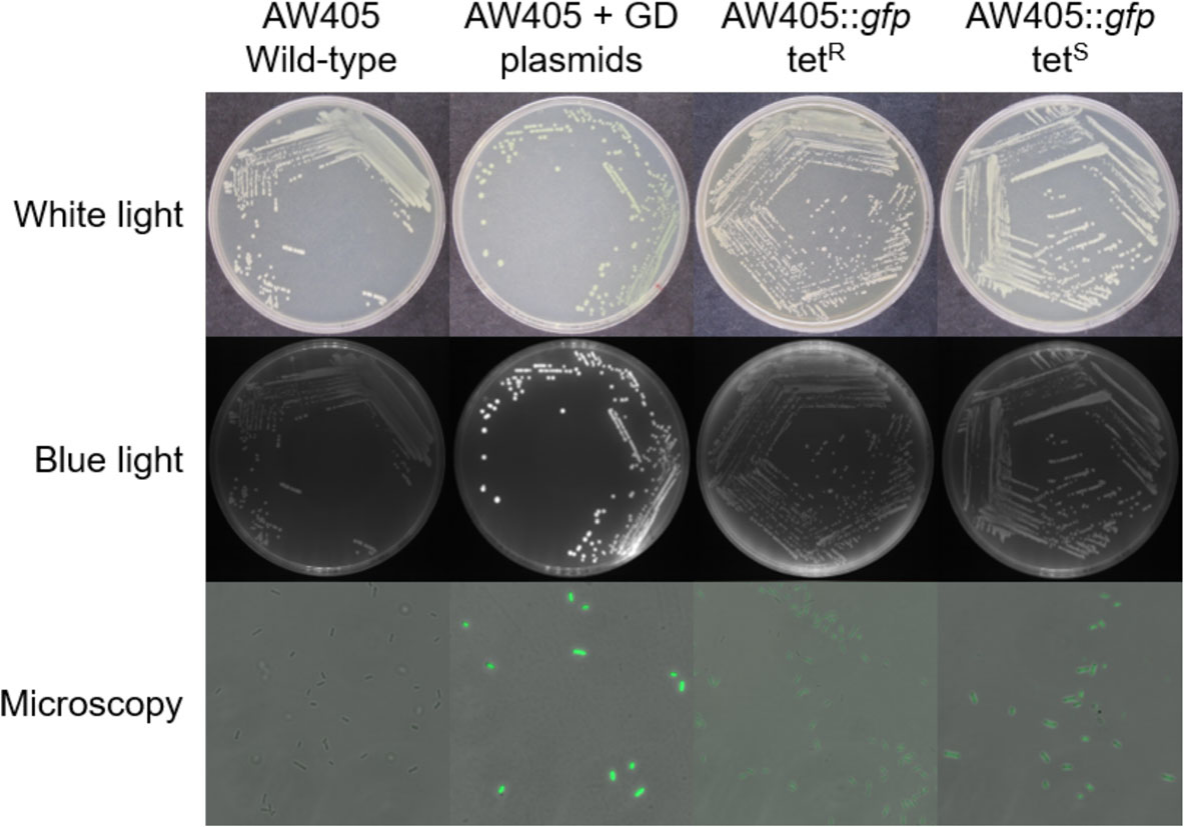
Comparison of cell phenotypes at key stages of the Gene Doctoring procedure to insert *sfgfp*. Fluorescence microscopy images are overlays of phase contrast and fluorescent images (488 nm excitation filter) at 1000× magnification

Successful colonies were white and did not noticeably fluoresce under blue light, both before and after removal of the tetracycline resistance cassette (Fig. 3). However, the cells fluoresced green when viewed by fluorescence microscopy, confirming the function of the chromosomal insertion. The reduction in green coloration for cells with *sfgfp* on the chromosome is due to the lower gene copy number compared to expression from a plasmid. We performed whole-genome shotgun sequencing on the final, tetracycline-sensitive cells to confirm the correct location and sequence of the *sfgfp* insertion, the absence of off-target recombination events and the loss of all plasmids (Supplementary Figure 2).

## Discussion

**Fig. 4:**
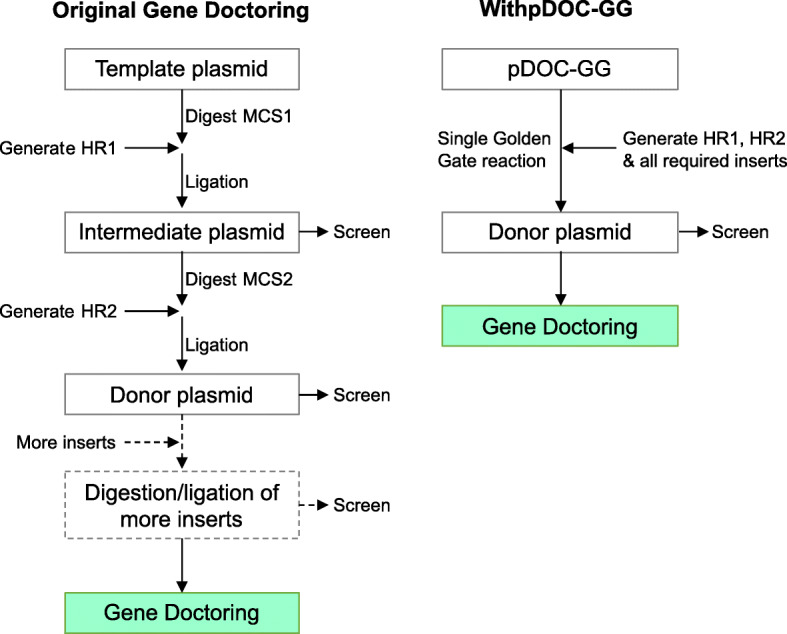
Comparison of the major steps in assembly of donor plasmids using the original Gene Doctoring plasmids and our pDOC-GG

Gene Doctoring is a convenient and efficient variation of recombineering, especially for non-domesticated and pathogenic strains of *E. coli*, which are often not amenable to transformation by the linear double-stranded DNA required by other methods. pDOC-GG harnesses Golden Gate assembly for single-reaction construction of donor plasmids, thus increasing the utility of Gene Doctoring by removing the number of cloning steps required to generate the donor plasmid, while retaining the advantages of the original system (Fig. 4). Our approach also theoretically allows a free choice of target sites, selection cassettes and type of modification (insertions, deletions, inversions, replacements and point mutations), while allowing integration with existing genetic part libraries, including those conforming to the popular MoClo molecular parts standard [17].

Although we have retained the original Flp recombinase-based technique for removal of the selectable marker in our experiments, scarless genome editing is possible using *tetA* as a counterselection marker [18]. Alternatively, any other selectable marker(s) can be used instead, to suit individual experimental requirements [19–23]. Regions HR1 and HR2 will vary between experiments and can easily be produced either as chemically synthesised DNA fragments, PCR products (with BsaI sites included on the primers) or from genetic parts libraries. We have made pDOC-GG publicly available, together with our amp^R^ version of mUAV and our genetic parts for inclusion of a *tetA* selection cassette on either side of an insertion (Table 1). We encourage further modification and sharing of these plasmids to broaden the available toolkit for Gene Doctoring of diverse targets and species with minimal effort.

**Table 1:** Genetic tools constructed during this study and made publicly available

Plasmid	Description	Addgene accession #
pDOC-GG	Acceptor plasmid for Golden Gate-style cloning of gene fragments to generate any Gene Doctoring donor plasmid in a single reaction.	149377
mUAVbla	Derivative of the universal acceptor vector from the Mobius Assembly toolkit [12], conferring an ampicillin resistance phenotype rather than chloramphenicol resistance.	149378
mUAVbla:tetR 5′	Gene part with a tetracycline resistance selectable marker flanked by FRT sites for BsaI-guided Golden Gate assembly. Removal from the chromosome will leave an FRT scar between homologous region 1 and an inserted sequence.	149379
mUAVbla:tetR 3′	Gene part with a tetracycline resistance selectable marker flanked by FRT sites for BsaI-guided Golden Gate assembly. Removal from the chromosome will leave an FRT scar between an inserted sequence and homologous region 2.	149380

## Materials and methods

### Bacterial strains

*E. coli* NEB5α (New England BioLabs product № C2987) or NEB10β (New England BioLabs product № C3019) cells were used as hosts during plasmid construction. Genome manipulations were performed on *E. coli* strain AW405 (a K-12 derivative), which was a gift from Howard Berg (Harvard University) [24].

### Competent cell production and transformation

*E. coli* competent cells were prepared following the CaCl_2_ method of Sambrook et al. [25] and transformed by heat-shock at 42 °C for 45 s followed by recovery in SOC medium.

### Construction of pDOC-GG

Beginning with pDOC-K, we engineered substantial modifications to produce a golden gate acceptor plasmid, named pDOC-GG, to enable single-step construction of donor plasmids. A 3308 bp DNA fragment containing the *sacB* expression cassette was amplified from pDOC-K by PCR with primers sacB_GA_F and sacB_GA_R (Fig. 1, Table 2) to add overlap regions for Gibson assembly. Four further fragments were then purchased as gBlocks from IDT (Leuven, Belgium). These fragments were a Kan^R^ cassette, the pMB1 and 5′ I-SceI recognition sequence, a *lacZα* reporter gene with flanking BsaI sites, and the 3′ I-SceI fragment (Fig. 1, Table 2). Each fragment was designed with appropriate sequences at each end to generate overlapping regions between neighbouring fragments for Gibson assembly. The Gibson assembly reaction contained 12 fmol of the *sacB* fragment, 10 fmol of the kanamycin resistance fragment, 50 fmol of the 3′ fragment and 20 fmol each of the remaining fragments in 5.5 μL, and an equal volume of 2× Gibson assembly master mix (NEB, Hitchin, UK). The reaction was incubated at 50 °C for 60 min, then 5 μL were used to transform NEB5α chemically competent cells, which were plated on LB agar containing kanamycin (50 μg.mL^− 1^), IPTG (1 mM) and X-gal (20 μg.mL^− 1^). Five blue colonies were selected and the plasmids were sequenced.

**Table 2:** Primers and chemically synthesized DNA fragments used in this study

Primer/fragment name	Sequence
**Primers**	
pDOC-K_F	CATGATTACGCCAAGCTCTAG
pDOC-K_R	GGGTTTTCCCAGTCACGACGT
sacB_GA_F	GGTAGTGTGGCGAGAGTAGGGAACTGCCAG
sacB_GA_R	CCAATTCTGACACATTTCCCCGAAAAGTGCC
J23106_FOR	CACCACAGGTCTCGACCTTTTACGGCTAGCTCAGTCC
sfGFPterm_REV	CACCACAGGTCTCGCGCTGTGACTCTAGTAGAGAGCG
Pbla_F_AatII	ATATATGACGTCGCGGAACCCCTATTTGTTTA
Pbla_R_SacI	ATATATGAGCTCTTACCAATGCTTAATCAGTGAG
mUAV_AmpR_screen_F	TGCATATAGAGACCGAATTCC
mUAV_AmpR_screen_R	CTTGGTCTGACAGCTCG
pBRforEco	AATAGGCGTATCACGAGGC
L4440	AGCGAGTCAGTGAGCGAG
glmS screen F	ACAAACGCATTGAAGCGCTG
glmS screen R	GAACCGATACCCTGGTAGTTA
**DNA fragments**	
pDOC_kan	ACTTTTCGGGGAAATGTGTCAGAATTGGTTAATTGGTTGTAACACTGACCCCTATTTGTTTATTTTTCTAAATACATTCAAATATGTATCCGCTCATGAGACAATAACCCTGATAAATGCTTCAATAATATTGAAAAAGGAAGAATATGAGCCATATTCAACGGGAAACGTCGAGGCCGCGATTAAATTCCAACATGGATGCTGATTTATATGGGTATAAATGGGCTCGCGATAATGTCGGGCAATCAGGTGCGACAATCTATCGCTTGTATGGGAAGCCCGATGCGCCAGAGTTGTTTCTGAAACATGGCAAAGGTAGCGTTGCCAATGATGTTACAGATGAGATGGTCAGACTAAACTGGCTGACGGAATTTATGCCACTTCCGACCATCAAGCATTTTATCCGTACTCCTGATGATGCATGGTTACTCACCACTGCGATCCCCGGAAAAACAGCGTTCCAGGTATTAGAAGAATATCCTGATTCAGGTGAAAATATTGTTGATGCGCTGGCAGTGTTCCTGCGCCGGTTGCACTCGATTCCTGTTTGTAATTGTCCTTTTAACAGCGATCGCGTATTTCGCCTCGCTCAGGCGCAATCACGAATGAATAACGGTTTGGTTGATGCGAGTGATTTTGATGACGAGCGTAATGGCTGGCCTGTTGAACAAGTCTGGAAAGAAATGCATAAACTTTTGCCATTCTCACCGGATTCAGTCGTCACTCATGGTGATTTCTCACTTGATAACCTTATTTTTGACGAGGGGAAATTAATAGGTTGTATTGATGTTGGACGAGTCGGAATCGCAGACCGATACCAGGATCTTGCCATCCTATGGAACTGCCTCGGTGAGTTTTCTCCTTCATTACAGAAACGGCTTTTTCAAAAATATGGTATTGATAATCCTGATATGAATAAATTGCAGTTTCATTTGATGCTCGATGAGTTTTTCTAACTGTCAGACC
pDOC_ori	GTTTTTCTAACTGTCAGACCAAGTTTACTCATATATACTTTAGATTGATTTAAAACTTCATTTTTAATTTAAAAGGATCTAGGTGTTTGATAATCTCATGACCAAAATCCCTTAACGTGAGTTTTCGTTCCACTGAGCGTCAGACCCCGTAGAAAAGATCAAAGGATCTTCTTGAGATCCTTTTTTTCTGCGCGTAATCTGCTGCTTGCAAACAAAAAAACCACCGCTACCAGCGGTGGTTTGTTTGCCGGATCAAGAGCTACCAACTCTTTTTCCGAAGGTAACTGGCTTCAGCAGAGCGCAGATACCAAATACTGTCCTTCTAGTGTAGCCGTAGTTAGGCCACCACTTCAAGAACTCTGTAGCACCGCCTACATACCTCGCTCTGCTAATCCTGTTACCAGTGGCTGCTGCCAGTGGCGATAAGTCGTGTCTTACCGGGTTGGACTCAAGACGATAGTTACCGGATAAGGCGCAGCGGTCGGGCTGAACGGGGGGTTCGTGCACACAGCCCAGCTTGGAGCGAACGACCTACACCGAACTGAGATACCTACAGCGTGAGCTATGAGAAAGCGCCACGCTTCCCGAAGGGAGAAAGGCGGACAGGTATCCGGTAAGCGGCAGGGTCGGAACAGGAGAGCGCACGAGGGAGCTTCCAGGGGGAAACGCCTGGTATCTTTATAGTCCTGTCGGGTTTCGCCACCTCTGACTTGAGCGTCGATTTTTGTGATGCTCGTCAGGGGGGCGGAGCCTATGGAAAAACGCCAGCAACGCGGCCTTTTTACGGTTCCTGGCCTTTTGCTGGCCTTTTGCTCACATGTTCTTTCCTGCGTTATCCCCTGATTCTGTGGATAACCGTACAGGAAACAGCTATGACCATGATTACGCCAAGCTCTAGGGATAACAGGGTAATCTACTGAGA
pDOC_lacZ	CAGGGTAATCTACTGAGACCTAATGTGAGTTAGCTCACTCATTAGGCACCCCAGGCTTTACACTTTATGCTTCCGGCTCGTATGTTGTGTGGAATTGTGAGCGGATAACAATTTCACACAGGAAACAGCTATGACCATGATTACGTCGGATTCACTGGCCGTCGTTTTACAACGTCGTGACTGGGAAAACCCTGGCGTTACCCAACTTAATCGCCTTGCAGCACATCCCCCTTTCGCCAGCTGGCGTAATAGCGAAGAGGCCCGCACCGATCGCCCTTCCCAACAGTTGCGCAGCCTGAATGGCGAATGGCGCCTGATGCGGTATTTTCTCCTTACGCATCTGTGCGGTATTTCACACCGCATATGGTGCACTCTCAGTACAATCTGCTCTGATGCCGCATAGTTAAGCCAGCCCCGACACCCGCCAACACCCGCTGACGCGCCCTGACGGGCGGTCTCAACGATAGGGATAA
pDOC_I-SceI	TCTCAACGATAGGGATAACAGGGTAATGAGCTTGGCACTGGCCGTCGTTTTACAACGTCGTGACTGGGAAAACCCTGGCGTTACCCAACTTAATCGCCTTGCAGCACATCCCCCTTTCGCCAGCTGGCGTAATAGCGAAGAGGCCCGCACCGATCGCCCTTCCCAACAGTTGCGCAGCCTGAATGGCGAATGGCGAGCTTGGCTGTTTTGGCGGATGAGAGAAGATTTTCAGCCTGATACAGATTAAATCAGAACGCAGAAGCGGTCTGATAAAACAGAATTTGCCTGGCGGCAGTAGCGCGGTGGTCCCACCTGACCCCATGCCGAACTCAGAAGTGAAACGCCGTAGCGCCGATGGTAGTGTGGCGAGAGTAGGGAACTGCC
TetR 3′ part 1	TCACCTGCATATCTCTCGCTGAAGTTCCTATACTTTCTAGAGAATAGGAACTTCGGAATAGGAACTTCTAGAACTTATCCTAATTTTTGTTGACACTCTATCATTGATAGAGTTATTTTACCACTCCCTATCAGTGATAGAGAAAAGTGAAATGAATAGTTCGACAAAGATCGCATTGGTAATTACGTTACTCGATGCCATGGGGATTGGCCTTATCATGCCAGTCTTGCCAACGTTATTACGTGAATTTATTGCTTCGGAAGATATCGCTAACCACTTTGGCGTATTGCTTGCACTTTATGCGTTAATGCAGGTTATCTTTGCTCCTTGGCTTGGAAAAATGTCTGACCGATTTGGTCGGCGCCCAGTGCTGTTGTTGTCATTAATAGGCGCATCGCTGGATTACTTATTGCTGGCTTTTTCAAGTGCGCTTTGGATGCTGTATTTAGGCCGTTTGCTTTCAGGGATCACAGGAGCTACTGGGGCTGTCGCGGCATCGGTCATTGCCGATACCACCTCAGCTTCTCAACGCGTGAAGTGGTTCGGTTGGTTAGGGGCAAGTTTTGGGCTTGGTTTAATAGCGGGGCCTATTATTGGTGGTTTTGCAGGAGAGATTTCACCGCATAGTCCCTTTTTTATCGCTGCGTTGCTAAATATTGTCACTTTCCTTGTGGTTATGTTTTGGTTCCGTGAAACCAAAAATACACGTGATAATACAGATACCGAAGTAGGGGTTGAGACGCAATCATATGCAGGTGT
TetR 3′ part 2	TCACCTGCATATAATCGAATTCGGTATACATCACTTTATTTAAAACGATGCCCATTTTGTTGATTATTTATTTTTCAGCGCAATTGATAGGCCAAATTCCCGCAACGGTGTGGGTGCTATTTACCGAAAATCGTTTTGGATGGAATAGCATGATGGTTGGCTTTTCATTAGCGGGTCTTGGTCTTTTACACTCAGTATTCCAAGCCTTTGTGGCAGGAAGAATAGCCACTAAATGGGGCGAAAAAACGGCAGTACTGCTCGGATTTATTGCAGATAGTAGTGCATTTGCCTTTTTAGCGTTTATATCTGAAGGTTGGTTAGTTTTCCCTGTTTTAATTTTATTGGCTGGTGGTGGGATCGCTTTACCTGCATTACAGGGAGTGATGTCTATCCAAACAAAGAGTCATCAGCAAGGTGCTTTACAGGGATTATTGGTGAGCCTTACCAATGCAACCGGTGTTATTGGCCCATTACTGTTTGCTGTTATTTATAATCATTCACTACCAATTTGGGATGGCTGGATTTGGATTATTGGTTTAGCGTTTTACTGTATTATTATCCTGCTATCGATGACCTTCATGTTAACCCCTCAAGCTCAGGGGAGTAAACAGGAGACAAGTGCTTAGTAAGCGGGACTCTGGGGTTCGAAATGACCGACGGCTCACCTTCGGGTGGGCCTTTCTGCGAGGAAGTTCCTATACTTTCTAGAGAATAGGAACTTCAGGTTGA GATATGCAGGTGT
TetR 5′ part 1	TCACCTGCATATCTCTGGAGGAAGTTCCTATACTTTCTAGAGAATAGGAACTTCGGAATAGGAACTTCTAGAACTTATCCTAATTTTTGTTGACACTCTATCATTGATAGAGTTATTTTACCACTCCCTATCAGTGATAGAGAAAAGTGAAATGAATAGTTCGACAAAGATCGCATTGGTAATTACGTTACTCGATGCCATGGGGATTGGCCTTATCATGCCAGTCTTGCCAACGTTATTACGTGAATTTATTGCTTCGGAAGATATCGCTAACCACTTTGGCGTATTGCTTGCACTTTATGCGTTAATGCAGGTTATCTTTGCTCCTTGGCTTGGAAAAATGTCTGACCGATTTGGTCGGCGCCCAGTGCTGTTGTTGTCATTAATAGGCGCATCGCTGGATTACTTATTGCTGGCTTTTTCAAGTGCGCTTTGGATGCTGTATTTAGGCCGTTTGCTTTCAGGGATCACAGGAGCTACTGGGGCTGTCGCGGCATCGGTCATTGCCGATACCACCTCAGCTTCTCAACGCGTGAAGTGGTTCGGTTGGTTAGGGGCAAGTTTTGGGCTTGGTTTAATAGCGGGGCCTATTATTGGTGGTTTTGCAGGAGAGATTTCACCGCATAGTCCCTTTTTTATCGCTGCGTTGCTAAATATTGTCACTTTCCTTGTGGTTATGTTTTGGTTCCGTGAAACCAAAAATACACGTGATAATACAGATACCGAAGTAGGGGTTGAGACGCAATCATATGCAGGTGT
TetR 5′ part 2	TCACCTGCATATAATCGAATTCGGTATACATCACTTTATTTAAAACGATGCCCATTTTGTTGATTATTTATTTTTCAGCGCAATTGATAGGCCAAATTCCCGCAACGGTGTGGGTGCTATTTACCGAAAATCGTTTTGGATGGAATAGCATGATGGTTGGCTTTTCATTAGCGGGTCTTGGTCTTTTACACTCAGTATTCCAAGCCTTTGTGGCAGGAAGAATAGCCACTAAATGGGGCGAAAAAACGGCAGTACTGCTCGGATTTATTGCAGATAGTAGTGCATTTGCCTTTTTAGCGTTTATATCTGAAGGTTGGTTAGTTTTCCCTGTTTTAATTTTATTGGCTGGTGGTGGGATCGCTTTACCTGCATTACAGGGAGTGATGTCTATCCAAACAAAGAGTCATCAGCAAGGTGCTTTACAGGGATTATTGGTGAGCCTTACCAATGCAACCGGTGTTATTGGCCCATTACTGTTTGCTGTTATTTATAATCATTCACTACCAATTTGGGATGGCTGGATTTGGATTATTGGTTTAGCGTTTTACTGTATTATTATCCTGCTATCGATGACCTTCATGTTAACCCCTCAAGCTCAGGGGAGTAAACAGGAGACAAGTGCTTAGTAAGCGGGACTCTGGGGTTCGAAATGACCGACGGCTCACCTTCGGGTGGGCCTTTCTGCGAGGAAGTTCCTATACTTTCTAGAGAATAGGAACTTCCGCTTGAGATATGCAGGTGT
*E. coli glmS* HR1	GGTCTCGCTACCGCGCTGGAAGGCGCATTGAAGTTGAAAGAGATCTCTTACATTCACGCTGAAGCCTACGCTGCTGGCGAACTGAAACACGGTCCGCTGGCGCTAATTGATGCCGATATGCCGGTTATTGTTGTTGCACCGAACAACGAATTGCTGGAAAAACTGAAATCCAACATTGAAGAAGTTCGCGCGCGTGGCGGTCAGTTGTATGTCTTCGCCGATCAGGATGCGGGTTTTGTAAGTAGCGATAACATGCACATCATCGAGATGCCGCATGTGGAAGAGGTGATTGCACCGATCTTCTACACCGTTCCGCTGCAGCTGCTGGCTTACCATGTCGCGCTGATCAAAGGCACCGACGTTGACCAGCCGCGTAACCTGGCAAAATCGGTTACGGTTGAGTAATAAATGGATGCCCTGCGTAAGCGGGGGGAGGGAGACC
*E. coli glmS* HR2	GGTCTCGAGGTCATTTTTCTTCCTGTTATGTTTTTAATCAAACATCCTGCCAACTCCATGTGACAAACCGTCATCTTCGGCTACTTTTTCTCTGTCACAGAATGAAAATTTTTCTGTCATCTCTTCGTTATTAATGTTTGTAATTGACTGAATATCAACGCTTATTTAAATCAGACTGAAGACTTTATCTCTCTGTCATAAAACTGTCATATTCCTTACATATAACTGTCACCTGTTTGTCCTATTTTGCTTCTCGTAGCCAACAAACAATGCTTTATGAATCCTCCCAGGAGACATTATGAAAGTTATGCGTACCACCGTCGCAACTGTTGTCGCCGCGACCTTATCGATGAGTGCTTTCTCTGTGTTTGCAGAAGCAAGCCTGACAGGTGCAGGTGCAACCTTCCCTGCGCCGGTGTATGCCAAATGGGACGAGGAGACC

### Cloning of genetic parts

The genes for sfGFP and Tet^R^ were cloned into plasmids to create genetic parts for Golden Gate assembly. The *sfgfp* gene was amplified together with its promoter and terminator by PCR using Q5 DNA polymerase (NEB, Hitchin, U.K.) and primers J23106_FOR and sfGFPterm_REV (Table 2) according to the manufacturer’s instructions. The template was plasmid pMA_Level2A [12]. The primers were designed to add BsaI sites for 5′ AGGT and 3′ CGCT overhangs, which caused *sfgfp* to be inserted in the reverse orientation relative to *glmS*. The PCR product was gel purified and then cloned into pMiniT 2.0 using a PCR cloning kit (NEB, Hitchin, U.K.) with NEB10β competent cells according to the manufacturer’s instructions. The tetracycline resistance cassettes were cloned into an ampicillin-resistant variant of plasmid mUAV from the Mobius Assembly Vector Toolkit (Addgene kit #1000000134) [12]. The β-lactamase (*bla*) gene was amplified by PCR from pMiniT 2.0 using primers Pbla_F_AatII and Pbla_R_SacI (Table 2), digested with AatII and SacI, and ligated to mUAV linearized with the same enzymes. Correct constructs were identified by colony PCR with primers mUAV_AmpR_screen_F and mUAV_AmpR_screen_R (Table 2). The new plasmid was named mUAVbla. The tetracycline resistance cassettes were each synthesized as two halves (Table 2) with AarI recognition sites to ligate them into mUAVbla as a full coding sequence, complete with promoter, terminator and flanking FRT sites, in a Golden Gate reaction, according to Andreou & Nakayama, [12] resulting in mUAVbla:tetR 5′ and mUAVbla:tetR 3′. Plasmids containing *tetA* were identified phenotypically and by colony PCR using primers pBRforEco and L4440 (Table 2), and the sequences of the plasmids were verified before use.

### Golden Gate assembly of gene doctoring donor plasmids

Donor plasmids were created from pDOC-GG in a Golden Gate assembly reaction using 10 fmol each of pDOC-GG and pre-cloned DNA sequences and 20 fmol of chemically synthesized sequences, 1 μL T4 DNA ligase buffer, 200 μg.mL^− 1^ bovine serum albumin, 0.5 μL T4 DNA ligase (NEB, Hitchin, U.K.) and 0.5 μL BsaI-HF v2 (NEB, Hitchin, U.K.) in a 10 μL reaction. The reaction consisted of 30 cycles of 37 °C for 3 min and 16 °C for 4 min followed by an incubation at 50 °C for 5 min and finally at 80 °C for 5 min. NEB5α cells were transformed using 2 μL of the reaction mix and white colonies growing on LB agar containing kanamycin (50 μg.mL^− 1^), tetracycline (10 μg.mL^− 1^), IPTG (500 mM) and X-gal (20 μg.mL^− 1^) were picked for screening.

### Gene doctoring

For induction of chromosomal integrations, the strain to be modified was transformed with the pDOC-GG-derived donor plasmid and the pACBSCE helper plasmid carrying the λ-Red genes. A single colony carrying both plasmids was grown in 500 μL of Miller’s LB medium with appropriate antibiotics at 37 °C for 4 h. The cells were pelleted and washed three times in filter sterilized 0.1× LB. They were then resuspended in 500 μL of 0.1× LB supplemented with 0.5% arabinose and incubated at 37 °C for 2–3 h for induction. 100 μL were plated on LB plates supplemented with tetracycline (10 μg.mL^− 1^) and 5% sucrose. The plates were incubated at room temperature for 48–72 h. Single colonies were checked for *sfgfp* insertion by colony PCR with primers glmS screen F and glmS screen R, annealing either side of the region to be modified. Absence of Gene Doctoring plasmids was checked by patching colonies to LB agar plates containing appropriate antibiotics and an index plate containing no antibiotic.

### Antibiotic resistance cassette removal

For the removal of the tetracycline resistance cassette, AW405::*tetA*-*sfgfp* was transformed with plasmid pCP20, carrying the genes for Flp recombinase activity, [13] and recovered at 30 °C on LB agar plates supplemented with carbenicillin (50 μg.mL^− 1^). Antibiotic cassette removal was confirmed by colony PCR with glmS screen F and glmS screen R, and by patching colonies to LB agar plates with and without tetracycline (10 μg.mL^− 1^) The antibiotic-sensitive clones were sub-cultured on LB agar at 42 °C to remove pCP20, which has a temperature-sensitive origin of replication. Absence of all plasmids was confirmed by testing the colonies’ sensitivity to all relevant antibiotics and the final modified strain was verified by whole-genome shotgun sequencing.

### Whole genome and whole plasmid shotgun sequencing

Genomic DNA was purified using a FastDNA Spin Kit for feces (MP Bio, Santa Ana, CA, U.S.A.) according to the manufacturer’s instructions, except that the final elution was in 200 μL of DNAse-free water rather than 60 μL. Plasmids were purified with a NucleoSpin Plasmid Miniprep kit (Macherey-Nagel, Düren, Germany). The DNA was quantified using a Quant-iT dsDNA high sensitivity assay kit (Thermo Fisher, Waltham, MA, U.S.A.) and normalised to 0.2 ng.μL^− 1^ in 10 mM Tris-HCl. Sequencing libraries were prepared with the Nextera XT DNA Library Prep kit (Illumina, San Diego, CA, U.S.A.). Libraries were quantified using the Quant-iT dsDNA high sensitivity assay kit. Genome samples were pooled in equal quantities, while plasmid libraries were first pooled together and the whole plasmid pool was added to the genomic pool at one-tenth the quantity of a genomic sample. The final pool was then run at a concentration of 1.8 pM on an Illumina NextSeq 500 instrument using a mid-output sequencing kit for 150 bp paired-end reads. Sequences were quality checked by FastQC v0.11.7 and trimmed with Trimmomatic v0.36 with a minimum read length of 40 bp and a sliding window of 4 bp with average quality of 15. The reads were then mapped against the expected sequence for each sample in Geneious Prime 2019 with the Geneious mapper and default settings.

## Supplementary information

**Additional file 1.** Supplementary Figures 1 and 2 as referenced in the manuscript.

## Data Availability

All data generated or analysed during this study are included in this published article. The full sequences of the plasmids constructed for this study are available in the Addgene plasmid database under the accession numbers given in Table 1 and the plasmids are available from Addgene on request.

## Abbreviations

HR1/2: Homologous Region 1/2
X-gal: 5-bromo-4-chloro-3-indolyl-β-D-galactopyranoside
IPTG: Isopropyl β-D-1-thiogalactopyranoside
PCR: Polymerase Chain Reaction
