# Supplementary material for: Creation of Golden Gate constructs for gene doctoring
Source: BMC Biotechnol. 2020 Oct 7;20:54. doi: 10.1186/s12896-020-00648-5 (PMC7542709; doi:10.1186/s12896-020-00648-5)
Supplement: Supplementary file 1 — Additional file 1. Supplementary Figures 1 and 2 as referenced in the manuscript. [file 12896_2020_648_MOESM1_ESM.pdf]

Creation of Golden Gate constructs for Gene Doctoring

Supplementary Figures

Authors: Nicholas M. Thomson[1], Chuanzhen Zhang[1,2,3], Eleftheria Trampari[1] and Mark J. Pallen[1,4,5*]

Affiliations:

[1]Quadram Institute Bioscience, Norwich Research Park, Norwich, Norfolk, NR4 7UQ, U.K.

[2]National Risk Assessment Laboratory for Antimicrobial Resistance of Animal Original Bacteria, College of Veterinary Medicine, South China Agricultural University, Guangzhou, 510642, China

[3]Guangdong Key Laboratory for Veterinary Drug Development and Safety evaluation, College of Veterinary Medicine, South China Agricultural University, Guangzhou, 510642, China

[4]School of Biological Sciences, University of East Anglia, Norwich Research Park, Norwich, Norfolk, NR4 7TU, U.K.

[5]School of Veterinary Medicine, University of Surrey, Daphne Jackson Road, Guildford, Surrey GU2 7AL, U.K.

[*]Corresponding author

mark.pallen@quadram.ac.uk

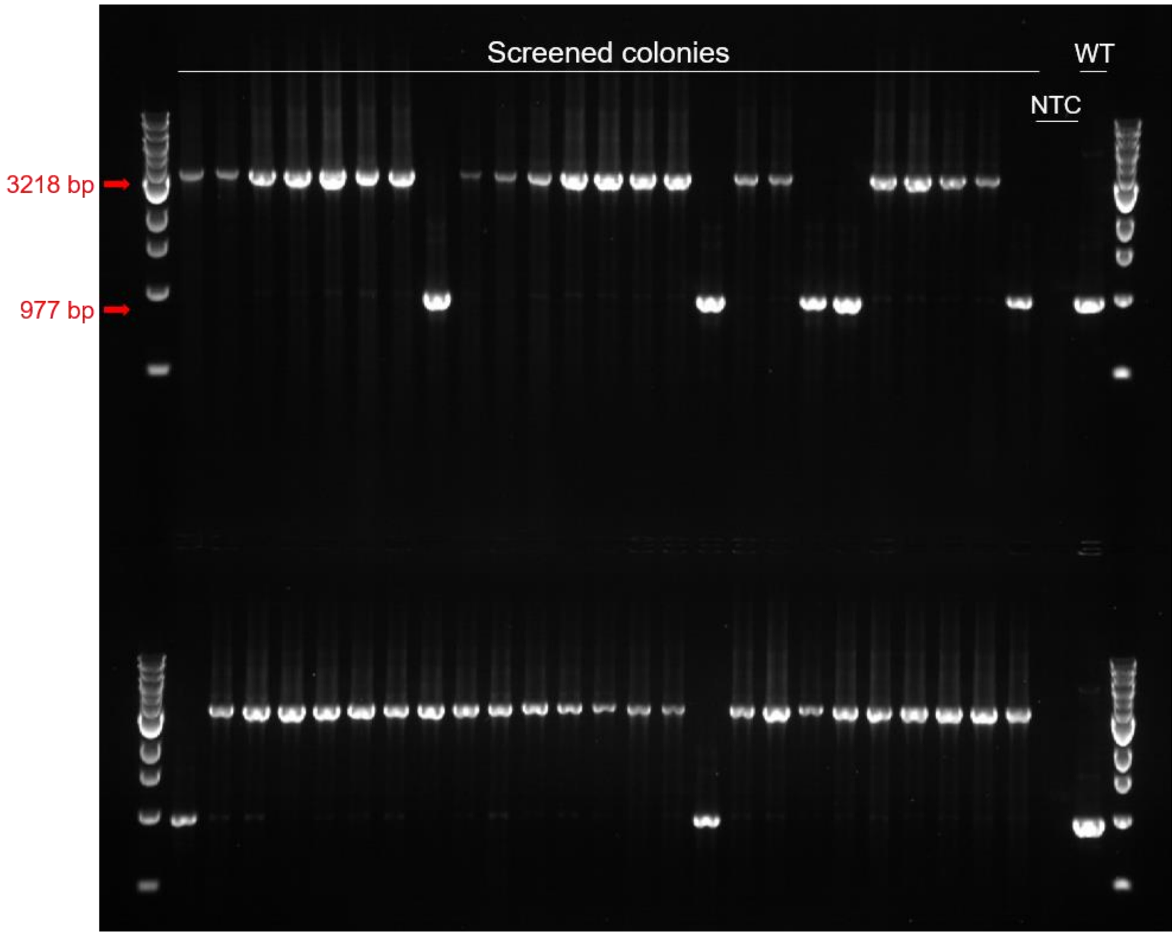

Supplementary figure 1: Agarose gel electrophoresis analysis of Gene Doctoring efficiency using a pDOC-GG-derived donor plasmid to insert *sfgfp* and a tetracycline resistance cassette into the *E. coli* AW405 chromosome. Following Gene Doctoring, cells were recovered on plates containing tetracycline (10 μg.mL^{-1}) and sucrose (5%). Fifty colonies were picked at random for colony PCRs using primers spanning the inserted region. Successful integration resulted in a 3,218 bp fragment. Cells with no integration gave 977 bp fragments. Each row of the gel was also loaded with 1 kb DNA ladder (first and last wells), a no template control (NTC) and a control reaction using wild-type AW405 (WT).

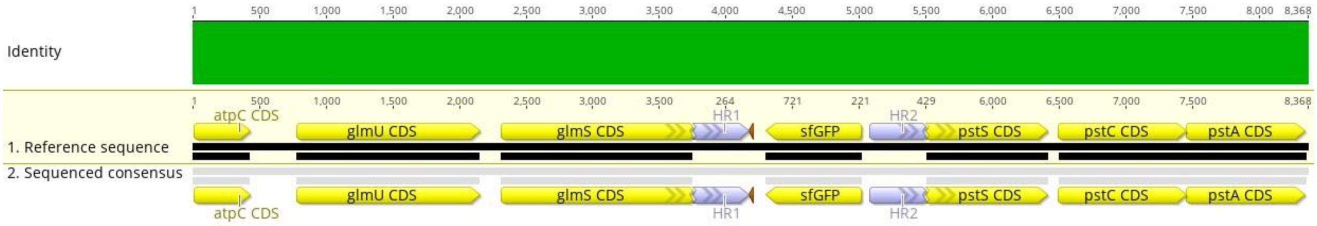

Supplementary figure 2: Confirmation of the insertion of *sfgfp* and subsequent removal the tetracycline resistance cassette in *E. coli* AW405. The final strain, which was fluorescent and sensitive to tetracycline, was sequenced by Illumina whole-genome shotgun sequencing. The reads were assembled into contigs and the *glmS* region was aligned against the expected (reference) sequence to confirm the success of the Gene Doctoring procedure. *sfgfp* and the surrounding genes are shown as yellow arrows, the homologous regions for recombination are indicated in purple and the FRT site scar from *tetA* removal is indicated by a brown arrowhead.
